# The prevalence and spectrum of mucocutaneous disease in South African people living with HIV and accessing care at a district-level hospital

**DOI:** 10.4102/sajhivmed.v21i1.1154

**Published:** 2020-12-10

**Authors:** Saskya Claasens, Susanna M.H. Kannenberg, Henry F. Jordaan, Karis Moxley, Rhodine Smith, Johann de Wet, Willem I. Visser

**Affiliations:** 1 Division of Dermatology, Department of Medicine, Faculty of Medicine and Health Sciences, Stellenbosch University, Cape Town, South Africa; 2 Research Development and Support Division, Faculty of Medicine and Health Sciences, Stellenbosch University, Cape Town, South Africa; 3 Division of Epidemiology and Biostatistics, Department of Global Health, Faculty of Medicine and Health Sciences, Stellenbosch University, Cape Town, South Africa

**Keywords:** mucocutaneous disease, HIV, district-level hospitals, ART, South Africa

## Abstract

**Background:**

Although the association between human immunodeficiency virus (HIV) and mucocutaneous diseases has been well studied within South African specialist centres, there is limited data from district-level hospitals. Available data may, therefore, fail to reflect the prevalence and full spectrum of dermatoses seen in people living with HIV (PLWH).

**Objectives:**

To determine the prevalence and spectrum of dermatoses seen in PLWH.

**Method:**

We conducted a cross-sectional, descriptive study of 970 PLWH (men and women, ≥ 18 years old) accessing care at Karl Bremer Hospital, a district-level hospital located in the Western Cape province, South Africa, between 01 September 2016 and 28 February 2017.

**Results:**

The prevalence of mucocutaneous disease in this sample was 12.7% (95% confidence interval [CI] 0.11–0.15). Non-infectious dermatoses comprised 71.0% of the disorders. Pruritic papular eruption (20.0%) and seborrheic dermatitis (6.0%) were the most common non-infectious dermatoses. Tinea corporis (8.0%) and oral candidiasis (6.0%) were the most prevalent infectious dermatoses. There was no significant association between skin disease category (infectious or non-infectious dermatoses) and patient demographics (gender and ethnicity) or HIV-disease characteristics (CD4+ cell count, viral load and duration of antiretroviral therapy [ART]).

**Conclusion:**

This study provides valuable scientific data on the prevalence and spectrum of mucocutaneous disease in PLWH attending a South African district-level hospital. Prospective studies conducted in other district-level centres across the country are required to determine the lifetime prevalence and spectrum of dermatoses in PLWH in the ART era.

## Introduction

The skin serves as an important clinical tool in the diagnosis and staging of patients with human immunodeficiency virus (HIV) and may be a marker of disease progression. Skin diseases are common and well described in the HIV-positive population and may affect up to 90% of individuals during the course of their illness.^1,2^ Although many skin and mucosal diseases are not unique to people living with HIV (PLWH), these patients can present with atypical features and dual or triple pathology.^3^ Furthermore, infective and inflammatory dermatoses in PLWH tend to be more severe and slower to respond to treatment when compared with the HIV-negative population.^4^

Antiretroviral therapy (ART) has altered the natural progression of HIV infection, leading to immune reconstitution through the suppression of viral replication and the recovery of the CD4+ cell count. As a result of ART, PLWH are living longer and healthier lives with a near-normal life expectancy.^5^ Several of the HIV-associated dermatoses have declined during the ART era, but certain drug reactions and inflammatory skin conditions have increased.^4,6^ Management of PLWH in the ART era includes addressing common non-infectious dermatoses in addition to rare opportunistic infections and infection-associated malignancies.^5^

Of the 7.2 million PLWH residing in South Africa, an estimated 61% were accessing ART in 2017.^7^ South African studies performed before the roll-out of ART described the cutaneous manifestations of HIV.^8,9,10^ Following the launch of ART, studies placed more emphasis on severe cutaneous adverse drug reactions and dermatoses that occur in the setting of tuberculosis (TB) co-infection.^11,12^ Although the existing body of literature provides valuable insights into dermatoses more likely to occur at advanced stages of immunosuppression, it does not sufficiently describe the full spectrum and prevalence of all mucocutaneous diseases occurring in PLWH. This is because most prior studies were performed at specialist and tertiary-level hospitals, and there is limited data available from district-level hospitals.

This limitation may have biased current knowledge and understanding regarding the spectrum and prevalence of dermatoses amongst PLWH. For example, studies performed at tertiary referral centres are more likely to include dermatoses that warrant inpatient management, such as inflammatory dermatoses, erythroderma and severe cutaneous drug reactions.^9,13^ Furthermore, most PLWH are cared for at primary and district-level healthcare facilities where they may present with common skin diseases that can be diagnosed and treated at the primary healthcare level. Overall, data from studies performed at tertiary referral centres could overestimate certain dermatoses and fail to represent the full spectrum of dermatoses seen in the majority of PLWH attending non-specialist centres.

This oversight may have important implications for the prescribed standard of care for PLWH at a district level. Therefore, to ensure that appropriate prevention and treatment strategies are implemented, there is a need to fully describe cutaneous diseases being treated at this level. Therefore, this study aimed to determine the prevalence and spectrum of HIV-related skin diseases at a South African primary healthcare facility. We additionally aimed to assess any possible associations between patient characteristics and dermatoses.

## Methods

### Study design

This descriptive and cross-sectional study was conducted at the adult HIV clinic at Karl Bremer Hospital (KBH), Cape Town, South Africa, between 01 September 2016 and 28 February 2017.

### Study setting

Karl Bremer Hospital is a publicly funded district-level hospital. It serves a heterogeneous patient population of low- to middle-income status. The HIV clinic is a division of the outpatient department and is operated by a nurse, two counsellors and a medical officer. Patients who require specialist dermatology care are referred to Tygerberg Hospital, which is a tertiary hospital in the vicinity.

### Study sample

All PLWH (men and women, ≥ 18 years old) seeking routine care at the HIV clinic at KBH were eligible to participate in the study. We included patients receiving ART and those awaiting ART commencement. We estimated an appropriate sample size using data provided in a previous study that reported the prevalence of cutaneous manifestations of HIV at a tertiary dermatology outpatient centre in KwaZulu-Natal (before the roll-out of ART).^10^ Herpes zoster was the most common cutaneous manifestation (19%) and opportunistic fungal infections were reported to be least prevalent conditions (3%). Using OpenEpi software (www.openepi.com), an expected prevalence of 10%, a 2% level of precision and a confidence interval (CI) of 95%, we calculated the required sample size to be 864. We considered this number to be sufficient to describe the spectrum of skin diseases in patients attending the HIV clinic with a high level of precision. During the 6-month study period, 970 patients were screened, of which 123 had skin disease and thus they were invited to participate in the study. The final sample comprised 100 patients who provided informed consent.

### Procedure

A data collection sheet was completed by the medical officer (author S.C.) working at the HIV clinic for all participants who signed informed consent. Data recorded included demographics, body mass index (BMI), recent (within the preceding 6 months) CD4+ cell count (recorded as either < 200 cells/*µ*L or ≥ 200 cells/*µ*L), viral load (recorded as undetectable or detectable) and ART regimen (type and duration). Antiretroviral therapy was defined as a regimen consisting of two nucleoside reverse transcriptase inhibitors (NRTIs) in combination with a non-nucleoside reverse transcriptase inhibitor (NNRTI) or a ritonavir-boosted protease inhibitor (PI).

The medical officer recorded a description of cutaneous disease found on routine clinical examination. Before the study commenced, the medical officer was trained to describe the duration, distribution and morphology of skin lesions of all participants in a standardised format. Where the dermatological diagnosis remained unclear after clinical examination, consent was obtained for taking the clinical photographs of skin lesions. Additional consent was obtained for the publication of clinical photographs. Clinical photographs were taken for a total of 45 participants. New cases were discussed weekly with dermatologists based at Tygerberg Hospital to verify the diagnosis of all skin conditions. To assist with diagnosis and management of the patient, skin biopsies were performed when it was clinically indicated.

The spectrum of skin diseases was broadly grouped into either infectious or non-infectious dermatoses. Infectious dermatoses were further divided into bacterial, viral and fungal infections and insect bite reactions. Dermatoses in the non-infectious group were separated into inflammatory, neoplastic, drug-related and other conditions. These characteristics were grouped based on clinically significant cut-off points and published data.^14,15,16,17,18,19,20^

### Data analysis

Continuous variables were summarised as mean and standard deviation (s.d.), whilst nominal variables were summarised as counts and percentages. Pearson’s chi-square test was used to assess the possible association between skin disease category and patient demographics (gender and ethnicity) or HIV disease characteristics (CD4+ cell count, viral load and duration of ART). Data were analysed using STATA (version 15; Stata Corp., College Station, Texas, USA) and statistical significance was set at *p* < 0.05.

### Ethical consideration

Ethical approval was obtained from the Health Research Ethics Committee of Stellenbosch University (N16/05/071). The study was also approved by the management of Karl Bremer Hospital. Participation was voluntary and all participants provided written informed consent to take part in the study. All data were anonymised to ensure the privacy and confidentiality of participants’ personal information and each participant was assigned a unique identifier.

## Results

We observed mucocutaneous disease in 123 patients out of a total of 970 who were screened, which is equivalent to a prevalence of 12.7% (95% CI 0.11–0.15). The demographic and clinical characteristics of the sample (*N* = 100 patients who provided informed consent) are summarised in Table 1. The mean age (± s.d.) was 40.4 (± 10.1) years. There were slightly more women (59%) than men (41%). Most participants were black people (75%), had a CD4+ cell count ≥ 200 cells/*µ*L (69%) and were on ART (74%). Of those participants on ART (*n* = 74), most (*n* = 65; 87.8%) were on an NNRTI-based regimen, whilst nine (12.2%) participants were on a PI-based regimen. Most patients (*n* = 45; 60.8%) had been on ART for more than a year. One-third of the sample (35%) had a normal weight (BMI 18.5–24), 24% were overweight (BMI 25–30) and 14% were obese (BMI > 30). Very few patients (7%) were classified as being underweight (BMI < 18.5). We were unable to calculate the BMI for 20 patients because of missing weight or height statistics in patient folders.

**TABLE 1:** Demographic and clinical characteristics of human immunodeficiency virus-positive patients with skin disease (*N* = 100).

Variable	*n*	%
Gender		
Male	41	41.0
Female	59	59.0
Ethnicity		
Black	75	75.0
Mixed ancestry	17	17.0
White	8	8.0
CD4+ cell count (cells/*µ*L)		
< 200	31	31.0
≥ 200	69	69.0
Viral load (copies/mL)		
Undetectable	52	52.0
Detectable	48	48.0
ART regimen		
Not taking ART	26	26.0
Taking ART	74	74.0
Duration of ART treatment†		
≤ 1 year	29	39.2
> 1 year	45	60.8

† , Percentages calculated relative to *n* = 74 patients who were taking ART at the time of the study.

ART, antiretroviral therapy; NNRTI, non-nucleoside reverse transcriptase inhibitor; PI, protease inhibitor.

A total of 121 dermatoses were identified in 100 study participants. The number of participants who presented with dual and triple pathology was 20 and 1, respectively. The spectrum of dermatoses in this sample is summarised in Table 2.

**TABLE 2:** Spectrum of dermatoses recorded for human immunodeficiency virus-positive patients (*N* = 100).

Dermatoses	*n*	%
Infectious dermatoses	50	50.0
Bacterial	8	8.0
Viral	14	14.0
Fungal	28	28.0
Non-infectious dermatoses	71	71.0
Inflammatory	50	50.0
Neoplastic	5	5.0
Drug related	2	2.0
Other	14	14.0

**FIGURE 1:**
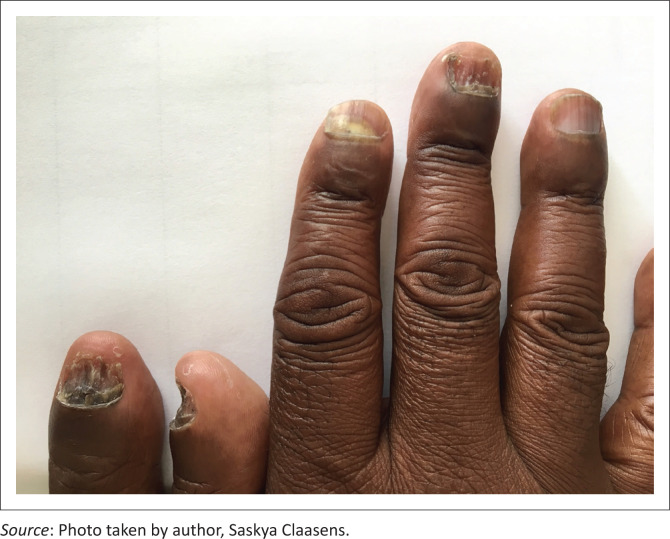
Proximal onychomycosis affecting four digits.

Infectious dermatoses affected half of the participants in the sample. Fungal infections were the most prevalent (*n* = 28, 28.0%), followed by viral (*n* = 14, 14.0%) and bacterial infections (*n* = 8, 8.0%). Fungal infections included oral candidiasis (*n* = 6), pityriasis versicolor (*n* = 4), intertriginous candidiasis (*n* = 1) and a spectrum of dermatophytosis (*n* = 17), namely, tinea corporis (*n* = 8), onychomycosis (*n* = 5) (Figure 1), tinea pedis (*n* = 3) and tinea faciei (*n* = 1). Overall, tinea corporis (8.0%) and oral candidiasis (6.0%) were the most prevalent of all infectious conditions. Bacterial infections included ecthyma (*n* = 3), folliculitis (*n* = 2), cellulitis (*n* = 1), impetigo (*n* = 1) and secondary syphilis (*n* = 1), whilst viral infections included condylomata acuminata (*n* = 4), herpes zoster (*n* = 2), molluscum contagiosum (*n* = 2), orolabial herpes simplex (*n* = 2), anogenital herpes simplex (*n* = 1), generalised verrucosis (*n* = 1), oral hairy leukoplakia (*n* = 1) and viral exanthem (*n* = 1).

**FIGURE 2:**
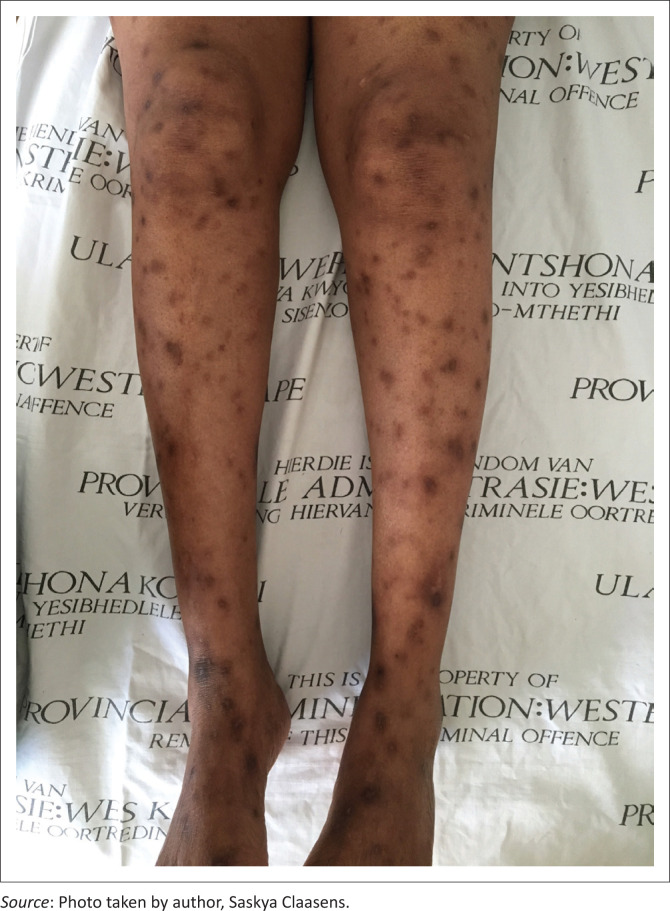
Pruritic papular eruption with post-inflammatory hyperpigmentation.

**FIGURE 3:**
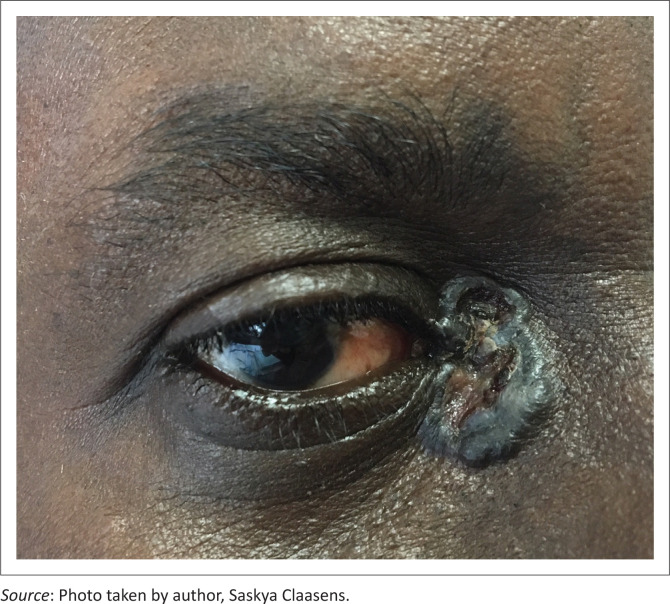
Pigmented basal cell carcinoma involving the medial canthus.

Non-infectious dermatoses affected 71.0% of the sample. Of the non-infectious dermatoses, inflammatory dermatoses were the most common (*n* = 50) and included pruritic papular eruption (PPE) (*n* = 20) (Figure 2), seborrheic dermatitis (*n* = 6), angular cheilitis (*n* = 4), atopic dermatitis (*n* = 4), contact dermatitis (*n* = 4), insect bite reaction (grouped urticarial papules on exposed sites such as the head, neck and distal extremities) (*n* = 4), dermatitis not otherwise specified (*n* = 3), chronic paronychia (*n* = 2), eosinophilic folliculitis (*n* = 2) and acne vulgaris (*n* = 1). Neoplastic conditions (*n* = 5) included actinic keratosis (*n* = 1), anal squamous cell carcinoma *in situ* (*n* = 1), basal cell carcinoma (*n* = 1) (Figure 3), cutaneous squamous cell carcinoma (*n* = 1) and seborrheic keratosis (*n* = 1). Drug-related dermatoses (*n* = 2) included steroid-induced rosacea (*n* = 1) and zidovudine-induced mucocutaneous hyperpigmentation (*n* = 1). Other dermatoses (*n* = 14) included post-inflammatory hyperpigmentation (*n* = 4), xerosis (*n* = 4), keloids (*n* = 3), vitiligo (*n* = 2) and palmoplantar keratoderma (*n* = 1).

Pearson’s chi-square test showed no significant association between skin disease category (infectious and non-infectious diseases) and patient demographics (gender, *p* = 0.36; ethnicity, *p* = 0.31; BMI, *p* = 0.61) or HIV disease characteristics (CD4+ count, *p* = 0.82; viral load [VL], *p* = 0.32; ART, *p* = 0.21; duration of ART, *p* = 0.28).

## Discussion

This study contributes novel information regarding the prevalence and spectrum of mucocutaneous diseases at a district-level hospital in South Africa. We estimated the prevalence of HIV-associated mucocutaneous disease to be 12.7%. Our search of the literature revealed that there appears to be a lack of South African and district-level data with which we can compare our findings. Nevertheless, our estimate is far below that recorded in other literature. Cross-sectional studies conducted in the ART era at specialist centres in China and South India recorded mucocutaneous disease in 62.9% and 69.41% of HIV-infected patients, respectively.^14,21^

This difference in prevalence estimates between specialist and district-level centres may be because of the impact of ART on mucocutaneous disease in the study population. Previous studies have found mucocutaneous disease to be less prevalent in patients who had received ART compared with those without ART.^14,17^ Calista et al. highlighted that a change in both the prevalence and type of cutaneous disorders is likely to be observed with the introduction of ART.^17^ This effect is likely because of immune restoration following sustained ART.

Kore et al. found that the proportion of HIV-infected patients having dermatoses increased with immunological worsening and that the average number of skin disorders per patient was significantly higher in patients with a CD4+ cell count less than 200 cells/*µ*L.^20^ Although we did not record the clinical stage of our study participants, the demographic data suggest a recovering immune profile in our study population. Most of our participants were taking ART (74%) and had a CD4+ cell count above 200 (69%) at the time of the study.

Non-infectious dermatoses were the most common group of mucocutaneous manifestations in our study. Inflammatory dermatoses were the predominant non-infectious dermatoses. Inflammatory dermatoses in the setting of HIV tend to be chronic, atypical and difficult to treat and, therefore, frequently warrant referral to specialist centres where the prevalence might be overestimated. This could explain why inflammatory dermatoses related to HIV (namely, seborrheic dermatitis, psoriasis, drug eruptions and erythroderma) were the most common dermatologic cause of admission in a South African study performed in the pre-ART era.^9^ However, studies in the ART era have persistently shown the spectrum of skin diseases shift from infectious to non-infectious dermatoses.^17,18,22^

In our study, the increased prevalence of inflammatory dermatoses was mainly attributed to PPE (20%) and seborrheic dermatitis (6%). The reported prevalence of PPE in the literature ranges from 11% to 46%.^5,23,24^ Two Ugandan studies have suggested that PPE may be related to an exaggerated immunological reaction to arthropod bites.^23,25^ The prevalence of PPE in our study may be attributed to its chronicity, its association with increased levels of immunosuppression (one-third of our study population had a CD4+ cell count below 200) and poor socio-economic conditions, which predispose patients to arthropod bites.^5,18,21^

Seborrheic dermatitis (6%) was the second most common inflammatory dermatosis in our study. The prevalence of seborrheic dermatitis has been reported to be 40% in HIV-positive patients and as high as 80% in acquired immunodeficiency syndrome (AIDS) patients in comparison with 3% in the HIV-negative population.^26^ Studies from China, India and the United States of America performed in the ART era have reported low prevalence rates of seborrheic dermatitis at 1.21%, 1.8% and 10.6%, respectively.^14,21,27^ Likewise, the prevalence of seborrheic dermatitis in our study is similar to that of the HIV-negative population and may be attributed to the influence of ART.

Neoplastic conditions were recorded in 5% of the study population. Since the introduction of ART, the incidence of AIDS-defining cancers (Kaposi’s sarcoma, non-Hodgkin’s lymphoma and cervical cancer) has decreased, whereas the relative frequency of non-AIDS-defining cancers has increased.^28^ Basal cell carcinoma and squamous cell carcinoma comprise the keratinocyte carcinomas and are the most common malignancies worldwide in both the general population and PLWH.^5,29^ The spectrum of neoplastic conditions observed in this study comprised benign epidermal neoplasms and keratinocyte carcinomas. Condylomata acuminata were observed in four patients, one of whom had concomitant anal squamous cell carcinoma *in situ*. Anal cancer (a human papillomavirus-related cancer) has been associated with the duration of both immunodeficiency and viral replication.^29^ As a result of increased life expectancy, cancer-specific screening programmes in HIV-infected patients should be extended to include non-AIDS-defining cancers such as anal cancer and keratinocyte cancer.

Drug-related dermatoses were observed in 2% of our study population and included steroid-induced rosacea and zidovudine (AZT)-induced mucocutaneous hyperpigmentation. In this study, no patients presented with severe cutaneous adverse drug reactions such as Steven–Johnson syndrome, toxic epidermal necrolysis or drug hypersensitivity syndrome. International studies in the ART era have reported a prevalence of drug eruptions between 10% and 17%.^14,17,18^ A study performed in a tertiary hospital in Cape Town, South Africa, found that most severe skin reactions resulting in admission occurred during the first 2 months after combination ART initiation. Nevirapine and pregnancy are also known to be strongly associated with severe skin reactions.^13^ Most of the participants receiving ART had been on treatment for more than 1 year and all those receiving an NNRTI-based regimen were taking efavirenz and not nevirapine. This could explain the absence of severe cutaneous adverse drug reactions in our study. None of the female participants was pregnant at the time of the current study. Lastly, patients presenting with severe skin reactions mostly necessitate inpatient management and referral to tertiary institutions and may not present directly to a district-level health facility.

Infectious dermatoses were observed in half of our study population and were encountered less frequently than non-infectious dermatoses. Tinea corporis (8%) and oral candidiasis (6%) were the two most common conditions in the infectious dermatoses group. Dermatophytosis (including tinea corporis, tinea faciei, tinea pedis and onychomycosis) is common in the setting of HIV and tends to occur as an early manifestation of immunosuppression at higher CD4+ cell counts.^30^ Dermatophytosis was recorded in 17% of our patients, which is in line with other studies performed in the ART era reporting a prevalence between 11.9% and 17.6%.^16,20,21^

Although oral candidiasis was one of the most common dermatoses recorded in our study, its prevalence is low compared with previous studies. The incidence of candidiasis has been shown to increase with a CD4+ cell count below 200.^14,15^ Kore et al. and Han et al. demonstrated a significant decline in candidiasis in their study population after the initiation of ART (20.8% vs. 11.5% and 64.04% vs. 16.6%).^14,20^ The majority of participants in our study were on ART and had a CD4+ count above 200. These factors, together with an increased awareness and early treatment of oral candidiasis, could likely explain its low prevalence.

The mean age of our study participants was 40 years, which is approximately 6 years older than that previously recorded in South African studies evaluating cutaneous manifestations of HIV in the pre-ART era.^9,10^ In general, PLWH in the ART era are increasingly older and have a near-normal life expectancy.^5,31^ Our findings are similar to those in a recent study performed at a specialised TB hospital in the Western Cape, which reported a mean age of 37 years in patients co-infected with TB and HIV.^12^ Historically, ART eligibility criteria in South Africa allowed for pregnant women to be initiated on ART at higher CD4+ cell counts compared with the general adult HIV-positive population.^32^ The routine surveillance of HIV during antenatal care may explain the increased proportion of females in this study as maternal services form an important referral pathway for ART services.

Our study has some limitations. Firstly, we emphasise that the accurate diagnoses of dermatoses can be challenging in HIV-positive patients because of the atypical presentation of skin disorders. Secondly, because of the cross-sectional nature of the study, the true prevalence of skin conditions in this study population may be underestimated. Long-term follow-up studies, as opposed to cross-sectional studies, are required to understand the true lifetime prevalence and spectrum of dermatoses in HIV-positive patients in the ART era. Thirdly, there could be an underestimation of dermatoses because none of the patients were examined by a dermatologist. Having a dermatologist see the patients primarily or using clinical photographs to verify the diagnosis of all skin conditions would have provided a more accurate presentation of dermatoses. Fourthly, our findings could be limited by self-report bias. Patients may be reluctant to mention minor skin complaints that do not interfere with daily functioning or cause cosmetic concern to avoid unnecessary time spent at the clinic.

## Conclusion

This study provides valuable scientific data on the prevalence and spectrum of mucocutaneous disease seen in PLWH attending a district-level hospital in South Africa. These findings could guide the development of training opportunities for primary healthcare workers to better manage the most prevalent HIV-associated dermatoses. This, in turn, may help to decentralise health services and improve the quality of care at primary and district levels. More studies performed outside tertiary-level hospitals are needed to provide insights into the true spectrum and prevalence of dermatoses affecting PLWH in the ART era.
